# Efficacy of Advanced Therapies as Prophylaxis and for Active Disease in Postoperative Crohn’s Disease: A Comprehensive Review

**DOI:** 10.3390/jcm14238435

**Published:** 2025-11-27

**Authors:** Atena Karimi, Alessandro David, Omar El Ouarzadi, Robert Battat

**Affiliations:** 1 Centre de Recherche du Centre Hospitalier de l’Université de Montréal (CRCHUM), Université de Montréal, Montreal, QC H2X 0A9, Canada; atena.karimi@umontreal.ca (A.K.); alessandro.david@umontreal.ca (A.D.); omar.el.ouarzadi@umontreal.ca (O.E.O.); 2 Division of Gastroenterology and Hepatology, Centre Hospitalier de l’ Université de Montréal (CHUM), Université de Montréal, Montreal, QC H2X 0C1, Canada

**Keywords:** Crohn’s disease (CD), postoperative recurrence (POR), advanced biologics, small molecules, therapeutic drug monitoring (TDM)

## Abstract

Postoperative recurrence (POR) in Crohn’s disease (CD) is common after intestinal resection, with over 50% developing endoscopic lesions within a year if untreated. The increasing availability of biologics and small molecules has transformed postoperative management, yet optimal strategies for prevention and treatment remain unclear. Infliximab and vedolizumab have the strongest evidence for preventing endoscopic recurrence in postoperative Crohn’s disease. Adalimumab and ustekinumab are viable alternatives supported by observational and post hoc trial data. Selective IL-23 inhibitors and JAK inhibitors have demonstrated high efficacy in moderate to severe luminal CD but lack dedicated postoperative trials. Personalized strategies, such as therapeutic drug monitoring (TDM), model informed dosing and pharmacogenetic profiling hold promise for improving long-term control of postoperative Crohn’s disease. Important gaps remain, particularly regarding the drug concentrations to target, the optimal timing for intervention, and the identification of patients most likely to benefit. Approaches that integrate disease location, clinical risk profiles, and knowledge of underlying immunopathogenic pathways could provide more precise clinical guidance. Finding molecular predictors of recurrence, directly comparing cutting-edge treatments, and integrating precision medicine techniques into standard postoperative care should be the main priorities of future research

## 1. Introduction

Crohn’s disease (CD) is a chronic, relapsing inflammatory bowel disease (IBD) that can affect any part of the gastrointestinal tract. Despite the advent of advanced medical therapies, approximately 30–50% of patients with CD will ultimately require intestinal surgery during their disease due to medically refractory disease or complications such as strictures, fistulas and abscesses [1]. However, surgery is not curative, and postoperative recurrence (POR) is common, with up to 80% of patients developing endoscopic recurrence within the first year postoperatively if no treatment is initiated [2]. After an initial resection, subsequent resections are often required (30%), despite the advent of advanced therapies [2]. The management of CD after surgery has evolved with the increasing availability of biologic and small molecule therapies. Traditionally, tumor necrosis factor antagonist agents such as infliximab and adalimumab have been used for both prophylaxis and treatment of POR, demonstrating efficacy in reducing endoscopic and clinical recurrence [3,4]. More recently, α4β7 integrin antagonists have shown a high level of evidence in preventing POR. Interleukin (IL)-12/IL-23 antagonists, IL-23 antagonists and Janus kinase (JAK) inhibitors have emerged, though their role in the postoperative setting remains less defined and supported by limited data.

In addition to pharmacological advances, strategies such as therapeutic drug monitoring (TDM), model-informed precision dosing, and the use of pharmacogenetics are shaping a more individualized approach to postoperative management. Nevertheless, significant knowledge gaps remain, including optimal drug selection, treatment duration, target drug levels, and the timing of intervention.

This review aims to critically examine the current evidence on the efficacy of advanced therapies in the prevention and treatment of postoperative CD, with a focus on biologics and small molecules. We also discuss the strategic use of these agents, the role of TDM, and emerging directions in personalized medicine.

## 2. Methods

A comprehensive search of the literature was performed on PubMed, MEDLINE and Embase databases up to July 2025. This search involved all peer-reviewed articles published in the English language. The search strategy included combinations of the following keywords: “Crohn”, “Crohn’s disease”, “CD”, “postoperative”, “recurrence”, “POR”, “biologics”, “anti-TNF”, “infliximab”, “adalimumab”, “certolizumab”, “ustekinumab”, “risankizumab”, “mirikizumab”, “guselkumab”, “anti-IL-23”, “anti-IL-12/23”, “vedolizumab”, ”anti-integrin”, “therapeutic drug monitoring”, “TDM”, “drug concentration”, “pharmacokinetics”, “immunogenicity”, “surgery”, “ileocecal resection”, “ICR”, “maintenance”, “treatment”, and “prophylaxis”. The reference lists of relevant reviews, studies, and clinical guidelines were manually screened to identify further eligible studies. Studies were included if they (1) involved adult patients with Crohn’s disease undergoing intestinal resection, (2) reported outcomes on POR prevention or treatment, (3) assessed biologics or small molecules, with or without therapeutic drug monitoring (TDM), and (4) were published in a peer-reviewed journal. Preference was given to randomized controlled trials (RCTs), observational cohorts, meta-analyses, and expert consensus documents published in the previous decade. Studies including the prevention, proactive and reactive TDM strategies and precision dosing approaches in the postoperative setting were also reviewed for eligibility We excluded pediatric studies, case reports, non-English publications, and studies not reporting specific postoperative outcomes.

Given that much of the evidence derives from observational cohorts rather than randomized controlled trials, we carefully considered potential confounding by indication—whereby higher-risk patients are preferentially prescribed certain therapies such as TNF antagonists or vedolizumab. When describing such studies, we emphasized whether statistical adjustment or propensity-score weighting was applied to account for baseline differences in patient risk profiles.

## 3. Pathophysiology and Risk Factors of POR in CD

POR in CD results from a multifactorial interplay involving immune dysregulation, microbial dysbiosis, genetic predisposition, and environmental risk factors that remain active after surgical resection.

### 3.1. Microbial Dysbiosis

Reduced abundance of *Faecalibacterium prausnitzii* has been consistently linked to POR [5]. Specifically regarding patients who have undergone ileocolonic resection, it has also been noted that higher primary bile acids correlate with inflammation in the ileum and significant modifications in gut microbiota, including decreased abundance of *F. prau* [6]. The anastomotic site itself and the neoterminal ileum are markedly vulnerable, allowing bile acids and luminal flow changes, bacterial overgrowth, and an altered mucosal barrier [7]. Interestingly, in a prospective study, endoscopic POR is associated with low alpha diversity, an enriched abundance of Proteobacteria, and decreased production of short-chain fatty acid producers such as *Lachnospiraceae* and *Ruminococcaceae*, consistent with alterations in microbes that resemble active ileal Crohn’s disease [8].

### 3.2. Immune Dysregulation

Memory T-lymphocytes, especially CD4^+^ effector memory T-cells that inhabit mesenteric lymph nodes (MLNs), play a pivotal role in POR. They maintain immunological memory and can migrate to the neoterminal ileum, where they reinstate mucosal inflammation. Chapuy et al., have shown that MLNs in patients with CD are enriched in Th17 memory T-cells, particularly effector memory Th17 (TEM) cells. Through an IL-12 dependent pathway, these cells can be reprogrammed into Th1-type cells within the MLN before trafficking back to the intestinal mucosa via specific homing signals, amplifying inflammation [9]. This positions the MLN as a key anatomical site of immune reactivation. Furthermore, clonal expansions of pathogenic T-cells in the mucosa have been correlated with recurrence risk. This immune cascade is sustained by elevated levels of proinflammatory cytokines such as IL-6 and TNF-α, while reduced expression of regulatory cytokines like IL-10 is also associated with early endoscopic recurrence [10,11].

### 3.3. Genetic Risk Factors

Several genetic variants have been implicated in the risk of POR in CD. Although NOD2 is a well-known CD susceptibility gene, its association with POR remains inconclusive, with a recent study identifying a specific polymorphism (rs2066844) as an independent predictor [12,13]. SMAD3, a gene unrelated to CD pathogenesis, has been associated with increased fibrosis and recurrence post-resection. Similarly, CARD8, a negative regulator of NF-κB and inflammasome activity, was identified as a potential predictor of surgical recurrence, although the underlying mechanisms remain unclear [12].

### 3.4. Clinical Risk Factors

Several clinical risk factors have been well established in the literature. These include smoking, prior intestinal resections, a penetrating disease phenotype, perianal disease, extensive disease location (particularly ileal involvement), younger age at diagnosis (<30 years), shorter disease duration (<10 years), and certain histologic features such as plexitis and granulomas at resection margins [14]. The surgical technique may also influence outcomes: stapled side-to-side anastomosis and more extensive mesenteric excision have been associated with lower recurrence rates in some studies [15]. These traditional risk factors have recently been challenged by a large, multicenter and multinational prospective cohort study that aimed to identify independent clinical predictors of endoscopic recurrence. In this study, the classical high-risk features, such as age <30 years, smoking, multiple prior surgeries, and penetrating disease with or without perianal involvement did not predict endoscopic recurrence at first colonoscopy. Instead, consistent predictors included male gender, non-White ethnicity, longer time between surgery and the first endoscopy, smoking after surgery, and the absence of TNF antagonist prophylaxis [16]. The discordance seen in recent studies may reflect the contribution of other factors, such as genetic susceptibility, microbial or immune features not captured by traditional clinical variables.

Identifying these risk factors is essential for stratifying patients based on their risk of postoperative recurrence. This risk assessment guides the selection of tailored therapeutic strategies.

### 3.5. Surgery as First-Line in Select Localized Disease

Multiple complications are associated with surgical interventions for CD as mentioned above; however, surgery remains a credible option even as a first-line therapy in select patients as highlighted by Agrawal et al. in a retrospective study. Historically, surgery was reserved for patients refractive to medical treatments or presenting with advanced, complicated disease. In this study including 1279 patients from Denmark, a comparison between early ileocecal resection (ICR) and anti-TNF therapy as a first-line treatment suggested better long-term outcomes for the ICR groups, with the risk of the composite outcome (more than 1 of: perianal disease, CD-related hospitalization, CD-related surgery and systemic corticosteroid exposure) being 33% lower with ICR [17]. Although this study is limited by observational biases, these results remain of interest as ICR can prevent morbidity and be cost-effective in specific cases. There is now a need for more high-quality studies to identify patients for whom it will be beneficial.

### 3.6. Infectious Complications After Surgery

While POR is the most described outcome in the post-operative context, infectious complications remain a leading cause of morbidity, ranging from surgical site infections (SSI) to intra-abdominal abscesses, anastomotic leaks, *Clostridioides difficile* infection (CDI), line-related infections and other nosocomial infections. While the type of surgery can affect the pre-operative risk of infection, there are identified host-variables that can be addressed. These modifiable risk factors include malnutrition, hypoalbuminemia, obesity, anemia, and the use of peri-operative transfusions [18]. Treatment prior to surgery with steroids has been associated with a higher risk of infection. Biologic agent use perioperatively does not increase risk of infection [19].

## 4. Definitions and Assessment of Postoperative Recurrence

POR in CD can be defined along a spectrum ranging from subclinical inflammatory changes to overt clinical relapse. Because symptoms often do not correlate with intestinal inflammation, recurrence must be evaluated using objective measures. POR can be classified across multiple axes.

### 4.1. Endoscopic Recurrence

Endoscopic POR is seen as mucosal lesions usually found at the neoterminal ileum or anastomosis, most commonly between 6 and 12 months after surgery. Endoscopic recurrence is typically assessed using the Rutgeerts score (RS), a validated index ranging from i0 (no lesions) to i4 (diffuse aphthous ileitis or large ulcers with nodules and narrowing). A score of i2 or higher is generally considered indicative of endoscopic recurrence, with i3 and i4 strongly predictive of clinical relapse [20]. Given the heterogeneity in the definition of the i2 subscore in the original RS, a modified classification system was later developed. Its improvement was meant to distinguish lesions localizing only within the ileocolic anastomosis (i2a) from those involving the neoterminal ileum (i2b), marked by the presence of greater than five aphthous ulcers separated by normal mucosa or by the presence of skip areas comprising larger lesions. Initial large multicenter study comparing the predictive ability of this modified Rutgeerts score (mRS) came up with strong evidence confirming in its place a positive correlation between an escalating mRS and a risk for surgical and clinical recurrence. Specifically, an index mRS ≥ i2b was independently linked with surgical recurrence, while an index mRS ≥ i1 correlated with clinical recurrence. In contrast, lesions limited to the anastomosis (i2a) were associated primarily with clinical recurrence only, suggesting that observation and close monitoring may be appropriate in these cases rather than immediate treatment escalation. Conversely, i2b lesions, reflecting neoterminal ileal involvement, generally prompt therapeutic intensification due to their higher risk of progression [21]. Further insights into i2a lesions have emerged from a Canadian study evaluating isolated anastomotic ulcers (IAUs) in the ileocolic anastomosis without neo-terminal ileum inflammation. It was demonstrated that IAUs are independently associated with a shorter time to POR, which underscores the potential value of assessing anastomotic and ileal lesions separately to improve prognostic accuracy. However, subsequent evidence has challenged this view [22]. A recent individual patient data meta-analysis pooling more than 400 patients found no major difference in recurrence risk between i2a and i2b lesions, even after adjusting for early initiation of biologic therapy [23]. This finding has reignited debate on whether both subgroups should be managed similarly rather than distinctly. As a result, clinical practice remains heterogeneous, some centers escalate therapy only for i2b or higher, while others intervene for any mRS ≥ i2 finding. These conflicting results highlight persistent uncertainty in how i2a lesions should be managed and emphasize the need for prospective studies to define standardized thresholds for treatment escalation.

### 4.2. Clinical Recurrence

Clinical recurrence is defined by the reappearance of gastrointestinal symptoms such as abdominal pain, diarrhea, or weight loss. In many studies, remission has been defined using the Harvey-Bradshaw Index (HBI) with a stringent threshold of <4. However, this index has not been validated in the postoperative setting, and the utility of symptoms for detecting recurrence of intestinal inflammation is limited by poor sensitivity and specificity [24]. The Crohn’s Disease Activity Index (CDAI) is one of the most popular clinical indices used to evaluate disease activity, with levels below 150 defining remission in clinical trials. However, the usefulness of the index in the postoperative setting is limited. In a cohort of 93 patients who underwent ileocolic resection, the index had a moderate correlation with symptomatic recurrence, with an optimal cut-off level of 148 or more, identifying active disease with 70% sensitivity and 81% specificity (AUC 0.78) in one of the studies. Despite this reasonable performance, the CDAI failed to capture asymptomatic endoscopic recurrence, which occurs in up to 20% of patients [25]. Another study showed no relation between symptom outcomes and endoscopic outcomes in post-operative CD [26]. Therefore, its role in predicting recurrence remains unproven, as endoscopic recurrence continues to be the main and most reliable predictive tool. Many patients with endoscopic recurrence remain asymptomatic, while others may experience symptoms from non-inflammatory causes, such as bile acid malabsorption. In fact, bile acid diarrhea (BAD) and fat malabsorption are recognized confounders that may resemble active CD, whereas the role of small intestinal bacterial overgrowth (SIBO) in disease activity scoring remains unestablished [7].

### 4.3. Non-Invasive Biomarkers

Accurate, non-invasive biomarkers for detecting POR are crucial for optimizing monitoring techniques and improving patient compliance in CD. While serological C-reactive protein (CRP) is commonly used but not very sensitive in the detection of early recurrence due to low disease burden, as well as the fact that few patients with active inflammation show elevated CRP levels, fecal calprotectin (FC) has gained prominence as more sensitive, with elevated levels (above 150 µg/g) being suggestive of early recurrence. However, thresholds postoperatively may tend to be lower, with serial monitoring every 4–6 months to guide timely endoscopy or therapeutic intensification [27]. The Endoscopic Healing Index (EHI; Monitr™ Panel) serves as an adjunct to classical markers by integrating 13 serum proteins yielding a validated score ranging from 0 to 100 that correlates with endoscopic findings. EHI still has the capacity to differentiate between remission and severe recurrence (≥i2) [21]. Serum leucine-rich α2-glycoprotein (LRG) is another endoscopy surrogate that has provided value in identifying intestinal inflammation [28]. Further biomarker discovery has also identified CXCL9, CXCL11, and MMP1 as robust predictors of postoperative recurrence. These involve the CXCR3 axis and innate immune pathways. Multi-marker serum panels containing these molecules are superior to the use of CRP alone when assessing for intestinal inflammation seen on colonoscopy [29]. Overall, these advances underscore the growing application of serum-based biomarkers for enhanced detection of recurrence, allowing risk-stratified surveillance and minimizing dependence on invasive tests in postoperative CD management.

### 4.4. Noninvasive Assessments

Radiologic modalities, including magnetic resonance enterography (MRE) and computed tomography enterography (CTE), can provide complementary information, particularly when endoscopy is contraindicated or when assessing for disease beyond the reach of the colonoscope. The sensitivity of enterography to detect endoscopic recurrence has ranged from 92 to 96% and its specificity from 75 to 88%. One study assessing the use of MRE in postoperative CD showed a sensitivity of 79%, specificity of 55%, positive predictive value of 68%, and negative predictive value of 68% [30]. No study yet has used these radiologic modalities to guide treatment. Intestinal ultrasound is increasingly utilized as a point-of-care modality, offering a non-invasive, cost-effective means of detecting bowel wall thickening and hyperemia indicative of inflammation. In a recent prospective study of 39 postoperative patients, IUS showed higher concordance with endoscopy than other non-invasive inflammatory biomarkers such as FC and CRP (κ = 0.50 vs. κ = 0.33) [31]. Video capsule endoscopy (VCE) has proven to provide a complementary, non-invasive alternative that allows for the direct visualization of the small bowel mucosa, boasting superior sensitivity for mucosal recurrence detection versus cross-sectional imaging. However, it is contraindicated in situations where there is known stenosis or fistula, owing to the risk of capsule retention [32]. Of interest, in a study by Han et al., when utilizing the information gleaned from VCE in managing the disease post-operatively, rates of clinical recurrence were significantly lower when compared to managing through ileocolonoscopy (2.7% versus 21.7%; *p* = 0.019) [33]. Moreover, Shiga et al. (2022) reported that repeated postoperative VCE identified residual or recurrent small-bowel activity in 85.7% of patients, and that treatment optimization based on capsule findings significantly decreased the risk of hospitalization, surgery, or dilation (HR, 0.45; HR for CE-guided, 0.30) [34]. The results confirm the usefulness of VCE in controlling CD in the outpatient setting, although larger studies will still be needed to assess its utility.

## 5. Advanced Therapies in Postoperative CD

Biologic and advanced therapies have transformed the management of CD, providing patients with a broader range of effective treatment options. Historically, thiopurines and TNF antagonists were recommended as prophylaxis for postoperative recurrence [2]. Over the past decade, newer classes of agents, including integrin antagonists, IL12/23 inhibitors, IL23 inhibitors, and Janus kinase (JAK) inhibitors, have been increasingly used after surgery. For newer classes, many postoperative CD studies are retrospective, often with small sample sizes and involving patients previously exposed to a TNF antagonist [35]. Importantly, the majority of available evidence has focused on their role in prophylactic therapy rather than in the treatment of established recurrence. The following section reviews each therapeutic class, summarizing key clinical trial data and comparative effectiveness.

### 5.1. TNF Antagonists

TNF antagonists, particularly infliximab and adalimumab, have shown efficacy for preventing recurrence after surgery. In the pivotal PREVENT trial, infliximab significantly reduced endoscopic recurrence (Rutgeerts score ≥ i2) at week 76 compared to placebo (30.6% vs. 55.5%; relative risk reduction 45%) in patients who had undergone ileocolonic resection. Symptom-based clinical recurrence was not statistically different between groups. Importantly, the PREVENT trial applied strict dosing with 5 mg/kg every 8 weeks starting within 45 days of surgery and showed good tolerability [36].

Further support for TNF antagonist efficacy comes from observational studies and meta-analyses. A prospective, open-label extension of a randomized controlled trial showed the long-term effect of infliximab. Patients initially randomized to infliximab or placebo for 1 year were followed for more than 5 years after ileocolonic resection. After the first year, treatment decisions were made at the discretion of the treating physician: patients originally on infliximab could either continue or stop therapy, while those initially on placebo could initiate infliximab or remain untreated. During the long-term follow-up, endoscopic recurrence was observed in only 22.2% of patients who received long-term infliximab, compared with 93.9% of those who did not receive infliximab (*p* < 0.0001) [29]. These results highlight the sustained protective effect of infliximab beyond the first postoperative year. In a network meta-analysis of 21 randomized controlled trials evaluating multiple therapies, TNF antagonist monotherapy significantly reduced the risk of clinical relapse (RR 0.04) and endoscopic relapse (RR 0.01) compared with placebo. TNF antagonists emerged as the most effective pharmacologic strategy for preventing POR, with large effect sizes relative to all other medications (clinical relapse RR 0.02–0.20; endoscopic relapse RR 0.005–0.04) [37].

Adalimumab has also shown superior efficacy over thiopurines in high-risk patients. In a subanalysis of the POCER randomized controlled study, endoscopic recurrence at 6 months occurred in 45% of patients receiving thiopurines compared to 21% in those treated with adalimumab (*p* = 0.028) [38]. Also, a randomized control trial on 51 patients showed the superiority of adalimumab to placebo, azathioprine, and mesalamine in preventing postoperative endoscopic and clinical recurrence in CD [39,40]. A retrospective multicenter study showed no significant difference in endoscopic recurrence rates at one year between infliximab and adalimumab (29% vs. 33%) [41]. Another multicenter retrospective study showed similar results [42].

These data establish TNF antagonists as the most effective strategy to prevent POR, with the potential to alter the natural course of CD after surgery [36,43,44].

### 5.2. Integrin Antagonist (Vedolizumab)

Vedolizumab (VDZ), a gut-selective integrin antagonist, has been evaluated as a prophylactic option for postoperative CD. The REPREVIO trial, a multicenter double-blind randomized controlled trial (RCT), assessed vedolizumab compared to placebo in high-risk patients treated within 4 weeks after surgery. At week 26, the probability of achieving a lower modified Rutgeerts score was 77.8% in the vedolizumab group (95% CI, 66.4–86.3; *p* < 0.0001). Severe endoscopic recurrence (mRS ≥ i2b) occurred in 23.3% of vedolizumab-treated patients versus 62.2% in the placebo group (*p* = 0.0004). In both groups, most patients had previous TNF antagonist exposure [45].

A retrospective multicenter study from the Spanish ENEIDA registry included 25 patients on vedolizumab and 40 patients on ustekinumab as postoperative prophylaxis. After a median follow-up of 17 and 26 months, the rate of endoscopic (40% vs. 42%) and clinical recurrence (32% vs. 30%) were similar [46]. Another retrospective analysis with prospective follow-up compared prophylactic postoperative vedolizumab (VDZ) with TNF antagonist for the prevention of POR. 81 patients were included; 22 were given VDZ and 59 were given TNF antagonists. Half of the patients in both arms had a history of intestinal surgery before, and 82% resumed preoperative biologic use; importantly, 91% of those who were on VDZ had prior exposure to TNF antagonists. Clinical remission and biochemical remission (CRP < 3 mg/L) at 6–12 months were comparable for VDZ and TNF antagonist (52% vs. 63%, *p* = 0.50; 50% vs. 62%, *p* = 0.43), respectively. Endoscopic remission (Simple Endoscopic Score for CD = 0) was lower in VDZ compared with TNF antagonist (25% vs. 66%, *p* = 0.01) [47].

### 5.3. Anti IL-12/23 (Ustekinumab)

Ustekinumab (UST), an anti-IL12/23 monoclonal antibody, has emerged as a promising agent for the prevention of POR in CD, particularly in patients previously exposed to a TNF antagonist.

There are no randomized controlled trials evaluating ustekinumab in the postoperative setting. However, several observational studies have demonstrated its potential efficacy in postoperative CD. Data from the ENEIDA registry showed results comparable to vedolizumab [46]. In a real-world multicenter European study, ustekinumab users had an endoscopic recurrence rate of 61.8% at one year, which was higher than that observed with TNF antagonists (40.2%) and vedolizumab (33%). However, after adjustment for confounders (such as prior surgery and biologic exposure), the risk of recurrence was not statistically different between groups [48]. In a large retrospective cohort including 186 patients, TNF antagonists achieved higher rates of deep remission (combining clinical and objective remission) than ustekinumab (44% vs. 16%, *p* = 0.008). In contrast, prophylactic efficacy appeared comparable between the two groups (47% vs. 35%, *p* = 0.4) [49].

In a retrospective cohort study by Buisson et al., including 63 postoperative CD patients, with 32 treated with ustekinumab and 31 with azathioprine, ustekinumab was significantly more effective than azathioprine in preventing endoscopic POR at 6 months. The recurrence rate (Rutgeerts ≥ i2) was 28.0% in the ustekinumab group versus 54.5% in the azathioprine group after inverse probability of treatment weighting (IPTW) (*p* = 0.029). A numerically lower rate of i2b recurrence was also observed (20.8% vs. 42.5%, *p* = 0.066) [50].

Ustekinumab appears to be a viable option for patients with prior TNF antagonist failure or contraindications to TNF antagonist. However, more prospective, controlled trials are needed to define its precise role relative to TNF antagonists and vedolizumab in first-line postoperative prophylaxis. A summary of observational studies and randomized controlled trials is provided in Table 1 and Table 2, respectively.

Vedolizumab is an effective prophylactic agent for recurrence in post-operative CD. However, limited retrospective data exists on agents other than anti-TNF and vedolizumab. No head-to-head randomized controlled trial has directly compared the vedolizumab and ustekinumab.

### 5.4. Other Advanced Therapy

Interleukin-23 (IL-23) inhibitors have emerged as promising therapeutic agents for CD, offering targeted immunomodulation through selective blockade of the IL-23p19 subunit. Risankizumab, mirikizumab, and guselkumab, have been shown to be effective and safe across induction, maintenance, and in head-to-head settings [58,59,60]. Despite their efficacy in CD, these agents have not been specifically investigated for the prevention of postoperative recurrence. More prospective studies are needed to include this agent as a formal indication for prophylaxis in postoperative CD.

Small molecules such as Janus kinase (JAK) inhibitors and sphingosine-1-phosphate (S1P) receptor modulators are also emerging as promising oral therapies for CD, offering alternatives to traditional biologic treatments. Among JAK inhibitors, upadacitinib has shown the most robust evidence for efficacy in moderate-to-severe CD [61,62]. Nonetheless, its specific role in the POR setting remains to be determined [63]. Ozanimod and etrasimod, both oral S1P receptor modulators, have demonstrated efficacy in ulcerative colitis and are under investigation for CD [64]. Their mechanism, involving lymphocyte trafficking modulation, may offer theoretical benefits in mucosal immune regulation post-resection, but clinical validation is lacking.

## 6. Treatment Strategies in Postoperative CD

### 6.1. Choice of Agents

Patients at high risk for POR should be initiated on advanced therapy for prophylaxis. These agents are also used for the treatment of recurrence. Selecting the optimal advanced therapy in the postoperative CD setting requires an individualized assessment of the patient’s prior treatment history, disease phenotype, comorbidities, and potential safety concerns [65]. Therapies previously associated with significant immunogenicity, or serious adverse events should generally be avoided. Primary or secondary loss of response should be interpreted with caution. Often patients are labeled as non-responders when therapy is initiated but CD has already progressed to a point in which surgery is required. It is also unclear whether pre-operative treatment failure mandates changing of medication class or whether the same treatment class can be re-initiated post operatively when no active disease remains.

### 6.2. Strategic Use for Prophylactic Purpose

While planning the initiation of the treatment of choice, there is a shown benefit in stratifying the patients based on their risk profile of POR. Commonly used risk-stratification markers such as penetrating disease were not shown to be strong predictors for endoscopic recurrence after accounting for cofactors and are now being questioned. When assessing for high-risk patients, emerging evidence points towards these factors: male gender, non-Caucasian ethnicity, smoking, prolonged time for a first postoperative endoscopy, and lack of TNF antagonists prophylaxis after resection [16]. For high-risk patients early initiation of biologic therapy (ideally within 2 to 4 weeks from surgery) is recommended over a delayed, endoscopy-guided approach [66]. This strategy aligns with the STRIDE-II recommendations, which emphasize that in CD, treatment goals should be based on objective markers of inflammation, namely endoscopic healing and normalization of FC and CRP rather than symptom control alone [67]. Such an approach represents a paradigm shift from reactive, symptom-driven management toward targeted, inflammation-based prophylaxis, ultimately reducing the likelihood of early endoscopic recurrence and future surgeries. The CALM trial, a phase 3 randomized study in patients with early CD, is further supportive of a treat-to-target strategy. By using a combination of clinical symptoms, CRP, and FC to guide the escalation of anti-TNF therapies, CALM showed superiority in clinical and endoscopic outcomes over treatment strategies driven by symptoms alone. Though not specifically in postoperative CD, this trial highlights the importance of monitoring via biomarkers in the prevention of disease progression. It is important that this paradigm be extrapolated to the postop era, where preemptive treatment prior to the onset of endoscopic recurrence can possibly be enabled. FC values of ≥150 µg/g of stool or a sustained elevation of ≥100 µg/g above the subject’s nadir in repeated analyses can help alert to early subclinical inflammation amenable to prompt therapeutic adjustment [68].

A colonoscopy at 6–12 months postoperatively should be performed to monitor for endoscopic recurrence and need for prompt treatment. In a cohort of 365 patients who underwent resection for CD, prophylactic TNF antagonists were shown to be protective for recurrence especially in patients who already received TNF antagonists prior to surgery [16]. Low-risk patients may benefit from a monitoring-based approach, with a colonoscopy planned 6 months postoperatively to assess endoscopic recurrence before the initiation of a biologic therapy. A proactive monitoring strategy was strongly supported by the POCER trial, which demonstrated that a scheduled colonoscopy at 6 months post-resection significantly reduced the rate of severe recurrence compared to a deferred colonoscopy at 18 months. Thus, performing an endoscopy routinely by 6 months subsequent to a surgery and adjusting therapy accordingly improves outcomes compared with relying on symptoms alone. Patients with recurrence, whether or not they received prophylactic therapy, should be started on advanced therapy [40]. This risk-stratification-based strategy aims to prevent irreversible bowel damage in high-risk patients while reducing overtreatment in low-risk patients. The therapeutic goal is to maintain mucosal healing. Indeed, Rutgeerts scores of i0–i2a are associated with an approximately 85% likelihood of remaining in clinical remission over two years and a low risk of requiring additional surgery, whereas scores of i2b–i4 indicate a higher risk of recurrence [69]. Patients with ≥i2b lesions should be treated with advanced therapy. For those already on advanced therapy, dose optimization or switching to a different mechanism of action is recommended. However, given the absence of head-to-head trials, treatment choice currently relies on the patient’s disease severity and phenotype, prior medication history, and comorbidities.

### 6.3. Strategic Use in Active Disease

Endoscopic recurrence proven with a colonoscopy 6 months postoperatively is a strong predictor of ongoing disease and thus requires prompt treatment. Among patients with an endoscopic recurrence, there is a significant benefit in initiating TNF antagonists shown both in a reduction in Rutgeerts score and prevention of further relapses. For patients who were already on TNF antagonists before recurrence, there is a benefit first in dose optimization and combination therapies [37]. Although TNF antagonists remains the treatment of choice, there is limited but growing evidence with ustekinumab, especially after failure of TNF antagonists, and vedolizumab to a lesser extent for treatment of recurrence after surgery [70].

### 6.4. Therapeutic Drug Monitoring in Postoperative CD

TDM is a key component in the strategic use of biologic therapies for postoperative prophylaxis. Proactive TDM involves scheduled monitoring to adjust drug levels regardless of symptoms to reduce treatment failure whereas reactive TDM involves monitoring when symptoms worsen to explain flare-ups and help adjust or switch biologic therapies [71]. After surgery, the aim is to prevent recurrence with early endoscopic assessment and initiation of therapy. TDM is increasingly used in this context to guide TNF antagonists. Proactive TDM aiming for predefined trough concentrations has been associated with improved outcomes, particularly in preventing immunogenicity and secondary loss of response [72]. Infliximab trough concentrations ≥3 µg/mL were historically considered the minimum to maintain remission, but recent evidence demonstrates infliximab ≥5 µg/mL at 6 months postoperatively to be safer in preventing recurrence [73]. However, there is still no strong consensus on target levels specific to the postoperative context. In a retrospective cohort on 130 patients, median serum levels assessed were 10 µg/mL for infliximab (IFX), 10.5 µg/mL for adalimumab (ADA), and 6.9 µg/mL for ustekinumab (UST). IFX levels of ≥3 µg/mL were associated with higher rates of deep (39% vs. 0%; *p* = 0.02), clinical (44% vs. 9%; *p* = 0.04), and objective remission (83% vs. 62%; *p* = 0.10) compared to lower levels. Similarly, ADA levels of ≥7.5 µg/mL were associated with higher deep (42% vs. 0%; *p* = 0.02), clinical (42% vs. 0%; *p* = 0.02), and objective remission (88% vs. 40%; *p* = 0.007). However, UST levels were not associated with remission outcomes [74]. A summary of proposed trough levels, monitoring times, and associated clinical decisions is given in Table 3 as a guide in optimizing anti-TNF therapies via TDM post-surgery.

We should note that the comparative benefit of resuming versus switching anti-TNF therapy in the postoperative setting remains unclear. Emerging data by Ahmed et al. suggest that surgical resection may alter the inflammatory and pharmacologic landscape, potentially restoring responsiveness to anti-TNF agents. Conversely, anti-TNF discontinuation preceding surgery is sometimes misclassified as treatment failure, complicating interpretation of postoperative outcomes.

## 7. Knowledge Gaps

The postoperative management of CD is a significant concern as bowel shortening predisposes the patient to recurrence. Although effective management strategies exist, there is a lack of critical care in the postoperative management of CD [40]. A crucial aspect of postoperative management that remains undefined is the use of TDM. Since optimal postoperative dosing of biologics is not well established, an important question is whether initiating a higher dose preoperatively could lead to adequate postoperative drug levels that help prevent recurrence [75]. As postoperative CD presents with reduced systemic inflammation, drug clearance may be less of a concern [76]. While therapies effective in CD are likely effective in prophylaxis against postoperative recurrence, the efficacy of other agents other than infliximab and vedolizumab are under explored [46,48,74].

## 8. Conclusions

CD recurrence after surgery continues to be a challenge in management. The advent of biologic therapies has led to a sustained reduction in repeat surgical resections, largely due to early postoperative initiation of biologics, enhanced monitoring through TDM and biomarkers, and advances in surgical techniques. Starting biologics earlier, intensive monitoring based on biomarkers, and more selectively performed bowel-sparing operations have reduced the necessity for repeat resection. Anti-TNF agents and vedolizumab are the best studied postoperative therapies, while ustekinumab, IL-23 antagonists and JAK inhibitors show encouraging retrospective data. Therapies effective in CD are likely effective in preventing its recurrence post-operatively. Early initiation within 2–4 weeks post-operatively, in combination with biomarker monitoring with TDM for TNF antagonists within 3 months and a colonoscopy at month 6 is the best strategy to reduce recurrence and react quickly if it occurs. A month 3 FC of 150 µg/g or higher or an elevated CRP level should trigger assessment or early endoscopy. This multidisciplinary, anticipatory approach defines the future of personalized postoperative care in CD.

## Figures and Tables

**Table 1:** Selected Observational Studies of Biologic Therapy for Prevention of Postoperative Recurrence in Crohn’s Disease.

Study Type	Drug Investigated	Number of Patients	Follow-Up Duration	Primary Endpoint	Endoscopic Response	Clinical Response	Reference
Retrospective Chinese Cohort	VED vs. UST vs. IFX	62	10 months	Clinical recurrence at the end of follow-up (CDAI > 150 and a CDAI increase of 100)	No significant difference	Clinical recurrence: 29.4% vs. 14.3% vs. 52.9%	Wang et al., 2025 [51]
Retrospective, multicenter study	UST vs. VED	65	31 months	Clinical and endoscopic recurrence	Endoscopic recurrence: 42% vs. 40%	Clinical recurrence: 32% vs. 30%	Mañosa et al., 2023 [46]
Retrospective study	UST	44	26 months	Reduction of at least one point in RS	50% achieved a reduction of at least one point in RS	72.7% clinical success	Macaluso et al., 2023 [52]
Retrospective multicentre study	TNF antagonist vs. VDZ vs. UST	297	3 years	Endoscopic recurrence rate RS ≥ i2 or SES-CD ≥ 6 at 1 year	Endoscopic recurrence: 40.2%, vs. 33% vs. 61.8% (*p* = 0.031)	Clinical recurrence: 62.0% vs. 78.1% vs. 60.9% (*p* = 0.201)	Yanai et al., 2022 [48]
Retrospective study	UST vs. TNF antagonist	186	12 months	Deep remission, defined by clinical and objective remission	Objective remission: 42% vs. 71% (*p* = 0.01)	Clinical remission: 40% vs. 61% (*p* = 0.08)	Ahmed et al., 2021 [49]
Retrospective multicenter study	UST vs. AZA	63	18 months	Endoscopic recurrence (RS≥ i2) at 6 months	Endoscopic recurrence: 28.0% vs. 54.5%, (*p* = 0.029)	Not mentioned	Buisson et al., 2021 [50]
Retrospective, multicenter study	IFX vs. ADA	179	54 months	Efficacy of TNF antagonist in achieving mucosal healing	Endoscopic response: 70% vs. 53% (*p* = 0.02) and endoscopic remission: 57% vs. 29% (*p* < 0.01)	No significant difference was observed between groups: clinical remission in symptomatic patients at anti-TNF initiation (61% vs. 57%) and clinical recurrence in asymptomatic patients with mucosal lesions (12% vs. 9%).	Cañete et al., 2020 [53]
Retrospective study	VDZ vs. TNF antagonist	80	12 months	Endoscopic recurrence	Endoscopic remission: 25% vs. 66% (*p* = 0.01)	No significant difference	Yamada et al., 2018 [47]

Abbreviations: AZA—Azathioprine, IFX—Infliximab, ADA—Adalimumab, UST—Ustekinumab, VDZ—Vedolizumab. TNF—Tumor necrosis factor, 6-MP—6-mercaptopurine, CDAI—Crohn’s Disease Activity Index, RS—Rutgeerts score.

**Table 2:** Recent Randomized Controlled Trials of Biologic Therapy for Prevention of Postoperative Recurrence in Crohn’s.

Drug Investigated	Number of Patients	Follow-Up Duration	Primary Endpoint	Endoscopic Recurrence	Clinical Recurrence	Reference
VDZ vs. placebo (REPREVIO trial)	84	18 months	Distribution of modified RS between treatment groups at week 26	modified RS ≥ i2b: 23.3% vs. 62.2% placebo (*p* = 0·0004)	12.5% vs.18.9%	D’Haens et al., 2025 [45]
ADA vs. 6-MP	35	1 Year	Endoscopic recurrence (RS ≥ i2) at 1 year	Week 32: 21% vs. 69% (*p* = 0.004); Week 58: 47% vs. 75% (*p* = 0.03)	Very low recurrence: 1 vs. 2	Hirsch et al., 2023 [54]
IFX vs. placebo	38	2 Years	Proportion of patients with endoscopic and/or clinical recurrence at 2 years after surgery.	Same rate for endoscopic and/or clinical recurrence 52.6% vs. 94.7% (*p* = 0.0032)	Same rate for endoscopic and/or clinical recurrence 52.6% vs. 94.7% (*p* = 0.0032)	Fukushima et al., 2018 [55]
ADA vs. AZA both with metronidazole	84	1 Year	Endoscopic recurrence at 1 year (RS ≥ i2)	42.2% vs. 59.0% (*p* = 0.12)	No significant difference	López-Sanromán et al., 2017 [56]
IFX vs. placebo	297	76 weeks	Endoscopic recurrence prevention	30.6% vs. 60% at week 76 (*p* < 0.002)	No significant difference in clinical recurrence: 12.9% vs.20% (*p* = 0.097)	Regueiro et al., 2016 [36]
Thiopurine/ADA vs. metronidazole alone	174	18 months	Endoscopic recurrence at 18 months (RS ≥ i2)	49% in active care vs. 67% in standard care	27% in the active care group had clinical recurrence (CDAI > 200) vs. 21 40% in standard care	De Cruz et al, 2015 [38]
ADA vs. AZA vs. mesalamine	51	2 years	Proportion of patients with endoscopic and clinical recurrence	6.3% vs. 64.7% vs. 83.3%	12.5% vs. 64.7% vs. 50%	Savarino et al., 2013 [39]
IFX vs. AZA	22	12 Months	Endoscopic, histological and clinical recurrence after 12 months of therapy	9% vs. 40% (*p* = 0.14)	No significant difference	Armuzzi et al., 2013 [57]

Abbreviations: AZA—Azathioprine, IFX—Infliximab, ADA—Adalimumab, UST—Ustekinumab, VDZ—Vedolizumab, SES-CD— Simple Endoscopic Score for Crohn’s Disease, CDAI—Crohn’s Disease Activity Index, RS—Rutgeerts score.

**Table 3:** Therapeutic Drug Monitoring (TDM) Targets and Recommended Clinical Actions in Postoperative Crohn’s Disease.

Checkpoint	Tumor Necrosis Factor Antagonist	Suggested Trough Concentration	Adjunct Marker	Interpretation/Likely Mechanism	Recommended Action
Month 3 (FCal + TDM)	Infliximab (IFX)	≥5 µg/mL	FCal < 150 µg/g	Optimal exposure; low inflammation	Continue regimen; Colonoscopic assessment at month 6
		<5 µg/mL + no antibodies	FCal ≥ 150 µg/g	Subtherapeutic drug level	Intensify (5 → 10 mg/kg or q6 wk)
		<5 µg/mL + antibodies	FCal ≥ 150 µg/g	Immunogenic loss	Add thiopurine (if prior ADA/IFX immunogenicity → strongly indicated) If high antibodies → switch drug
Month 3 (FCal + TDM)	Adalimumab (ADA)	≥8 µg/mL	FCal < 150 µg/g	Optimal target for prophylaxis	Maintain dose; Colonoscopic assessment at month 6
		<8 µg/mL + no antibodies	FCal ≥ 150 µg/g	Underexposure	Increase to weekly dosing
		<8 µg/mL + antibodies	FCal ≥ 150 µg/g	Immunogenic loss	Add thiopurine or consider switch drug
Month 6 (Scope + TDM)	IFX/ADA	optimal trough concentration + endoscopic remission	RS i0–i1	Sustained remission	Maintain; may extend interval
		optimal trough concentration + endoscopic recurrence	RS ≥ i2	Pharmacodynamic failure	Switch to an agent with a different mechanism of action

Abbreviations: FCal—Fecal calprotectin, RS—Rutgeerts score, q6w—Every 6 weeks.

## Data Availability

No new data were created or analyzed in this study.
